# *Helicobacter Pylori* Targets the EPHA2 Receptor Tyrosine Kinase in Gastric Cells Modulating Key Cellular Functions

**DOI:** 10.3390/cells9020513

**Published:** 2020-02-24

**Authors:** Marina Leite, Miguel S. Marques, Joana Melo, Marta T. Pinto, Bruno Cavadas, Miguel Aroso, Maria Gomez-Lazaro, Raquel Seruca, Ceu Figueiredo

**Affiliations:** 1 Ipatimup–Institute of Molecular Pathology and Immunology of the University of Porto, 4200-135 Porto, Portugal; miguel_s_marques@hotmail.com (M.S.M.); jmelo@ipatimup.pt (J.M.); mtpinto@ipatimup.pt (M.T.P.); bcavadas@ipatimup.pt (B.C.); rseruca@ipatimup.pt (R.S.); 2 i3S–Instituto de Investigação e Inovação em Saúde, Universidade do Porto, 4200-135 Porto, Portugal; miguel.aroso@i3s.up.pt (M.A.); maria.glazaro@ineb.up.pt (M.G.-L.); 3 Department of Pathology, Faculty of Medicine of the University of Porto, 4200-319 Porto, Portugal; 4 ICBAS–Instituto de Ciências Biomédicas Abel Salazar, Universidade do Porto, 4050-313 Porto, Portugal; 5 INEB–Instituto de Engenharia Biomédica, Universidade do Porto, 4200-135 Porto, Portugal

**Keywords:** *Helicobacter pylori*, EPHA2, receptor tyrosine kinases (RTKs), angiogenesis, invasion, cell–cell adhesion, cell–matrix adhesion, RTK therapy, SRC inhibitors, gastric cancer

## Abstract

*Helicobacter pylori*, a stomach-colonizing Gram-negative bacterium, is the main etiological factor of various gastroduodenal diseases, including gastric adenocarcinoma. By establishing a life-long infection of the gastric mucosa, *H. pylori* continuously activates host-signaling pathways, in particular those associated with receptor tyrosine kinases. Using two different gastric epithelial cell lines, we show that *H. pylori* targets the receptor tyrosine kinase EPHA2. For long periods of time post-infection, *H. pylori* induces EPHA2 protein downregulation without affecting its mRNA levels, an effect preceded by receptor activation via phosphorylation. EPHA2 receptor downregulation occurs via the lysosomal degradation pathway and is independent of the *H.* *pylori* virulence factors CagA, VacA, and T4SS. Using small interfering RNA, we show that EPHA2 knockdown affects cell–cell and cell–matrix adhesion, invasion, and angiogenesis, which are critical cellular processes in early gastric lesions and carcinogenesis mediated by the bacteria. This work contributes to the unraveling of the underlying mechanisms of *H. pylori*–host interactions and associated diseases. Additionally, it raises awareness for potential interference between *H. pylori* infection and the efficacy of gastric cancer therapies targeting receptors tyrosine kinases, given that infection affects the steady-state levels and dynamics of some receptor tyrosine kinases (RTKs) and their signaling pathways.

## 1. Introduction

*Helicobacter pylori* is a highly prevalent extracellular Gram-negative bacterium found worldwide that is not cleared by the host’s immune system and establishes a life-long infection in human gastric mucosa. *H. pylori* is classified as a class I carcinogen and is an etiological factor of gastric adenocarcinomas, also contributing to chronic gastritis, peptic ulcer disease, and gastric mucosa-associated lymphoid tissue lymphoma [1].

Chronic interaction between *H. pylori* and the human gastric epithelium continuously activates host signaling pathways, imprinting cellular and molecular alterations. Among the host signaling pathways known to be activated by *H. pylori* are those associated with receptor tyrosine kinases (RTKs) [2]. Particularly, *H. pylori* activates MET, a member of the hepatocyte growth factor receptor family, and members of the epidermal growth factor receptor (EGFR) family, to modulate critical host cellular processes, such as motility, migration, invasion, proliferation, apoptosis, and autophagy [3,4,5,6,7,8,9,10,11,12,13,14].

In humans, the largest family of RTKs comprises the erythropoietin-producing hepatocellular (EPH) receptors, which include fourteen receptors divided into two classes, namely, class A receptors with nine members (EPHA1–EPHA8 and EPHA10), and the class B receptors with five members (EPHB1–EPHB4 and EPHB6). These classes are defined according to their sequence homology and binding affinity to ephrins (EFN), their ligands [15]. Unlike other RTKs whose ligands are soluble, both EPH receptors and EFN ligands are membrane-anchored, enabling bi-directional signaling in both EPH- and EFN-expressing cells upon cell–cell contact. Structurally, EPH receptors comprise an extracellular region containing an N-terminal ligand-binding domain, a cysteine-rich region, and two fibronectin type III repeats. This is followed by a single transmembrane segment and a cytoplasmic domain with a short juxtamembrane segment, a tyrosine kinase domain, a sterile α-motif, and a PDZ-binding domain at the C-terminus region [15,16,17]. In a resting state, EPH kinase activity is autoinhibited. Upon activation through interaction with ephrin ligands, the phosphorylation of the tyrosine residues in the juxtamembrane region relieves the autoinhibition, allowing the kinase domain to adopt an active conformation and initiating downstream signaling [15,18,19].

EPH receptors are important mediators in a wide range of biological functions, such as cell adhesion, migration, invasion, and angiogenesis. They are also involved in several pathological conditions, including cancer, when their expression and/or function are deregulated [20,21,22,23,24,25,26,27,28,29]. EPHA2 is overexpressed at the mRNA or protein level in various types of solid cancers, both in cell lines and in primary tumor samples [30,31]. Overexpression of the EPHA2 receptor has been associated with epithelial-to-mesenchymal transition, metastasis, and poor prognosis of gastric cancer patients [32,33,34,35,36,37,38].

More recently, EPH family members were reported as targets of microbial pathogens, underscoring their relevance in host-cell infection and pathogenesis mechanisms. Specifically, the EPHA2 receptor is a host cofactor for Kaposi’s sarcoma-associated herpesvirus (KSHV) [39,40], and an entry receptor for Epstein-Barr virus (EBV) [41,42] and the obligate intracellular bacterium *Chlamydia trachomatis* [43]. EPHA2 functions as an epithelial cell pattern recognition receptor for β-glucans, in addition to being an entry receptor in *Candida albicans* [44]. So far, there are no published descriptions on the relationship between *H. pylori* infection and EPH receptors, apart from a tyrosine phosphoproteomic screening that detected tyrosine phosphorylation of the EPHA2 receptor upon infection of AGS cells [45].

In this study, we investigated the impact of *H. pylori* infection on the EPHA2 receptor using two different gastric cell lines, MKN74 and NCI-N87. Our findings provide evidence that *H. pylori* targets the EPHA2 receptor through a mechanism independent of the major virulence factors CagA, VacA, and type four secretion system (T4SS), and that long-term infection (after 16 h) induces a decrease in EPHA2 receptor protein levels without significantly changing its mRNA levels. EPHA2 receptor downregulation by *H. pylori* was preceded by receptor tyrosine and serine897 phosphorylation and was followed by degradation via the lysosomal pathway. Using small interfering RNA for the EPHA2 receptor, we demonstrated that the silencing of EPHA2 in gastric epithelial cells impaired cell–cell adhesion, cell–matrix interactions, invasion on Matrigel, and angiogenesis. Overall, our results indicated that *H. pylori* interferes with critical cellular functions via EPHA2 receptor targeting, which are probably important in early gastric lesions and gastric carcinogenesis prompted by the bacteria.

## 2. Materials and Methods

### 2.1. Antibodies, Pharmacologic Inhibitors, and Chemicals

The antibodies used in this study included rabbit polyclonal anti-AKT (#9272; Cell Signaling Technologies Inc., Danvers, MA, USA), rabbit polyclonal anti-phospho-Ser473-AKT (#4060; Cell Signaling Technology), rabbit polyclonal anti-EPHA2 (clone C-20; sc-924; Santa Cruz Biotechnology Inc., Dallas, TX, USA), rabbit monoclonal anti-EPHA2 (clone D4A2; #6997; Cell Signaling Technology), rabbit mAb phosphoSer897-EPHA2 (Clone D9A1; #6347; Cell Signaling Technology), rabbit pAb phospho Tyr772-EPHA2 (#8244; Cell Signaling Technology), mouse monoclonal anti-GAPDH (clone 0411; sc-47724; Santa Cruz), mouse monoclonal anti-alpha 1 integrin (clone SR84; #559594, BD Biosciences, San Jose, CA, USA), mouse monoclonal anti-beta 1 integrin (clone JB1B; sc-59829, Santa Cruz Biotechnology), rabbit polyclonal anti-p44/42 MAPK (ERK1/2; clone 137F5; #4695; Cell Signaling Technology), rabbit monoclonal anti-phospho Thr202/Tyr204-p44/42 MAPK (ERK1/2; clone D13.14.4E; #4370; Cell Signaling Technology), Alexa Fluor 488 goat anti-rabbit IgG (#A11034; Thermo Fisher Scientific, Waltham, MA, USA), rabbit polyclonal IgG (ab27478, Abcam, Cambridge, UK), mouse monoclonal antibody PY99 (sc-7020; Santa Cruz Biotechnology), mouse monoclonal anti-SRC (clone L4A1; #2110; Cell Signaling Technology), rabbit polyclonal anti-phospho Tyr416-SRC Family (#2101; Cell Signaling Technology), and mouse monoclonal anti-α-Tubulin (clone B-5-1-2; #T5168; Sigma-Aldrich Co., St. Louis, MO, USA). The pharmacological inhibitors used were U0126 (Cayman chemical, MI, USA), which is a mitogen-activated protein kinase (MAPK)/ERK kinase (MEK) inhibitor, *CAY10626* (Cayman), a dual phosphatidylinositol-3-kinase (PI3Kα) and mTOR inhibitor, and the SRC kinase family inhibitors PP2 (Cayman) and Dasatinib (Selleck Chemicals, Houston, TX, USA). Concanamycin A (Sigma-Aldrich) and Bafilomycin A1 (Calbiochem^®^ MerckMillipore, Darmstadt, Germany) were used as lysosomal inhibitors and bortezomib (S1013; Selleck) was used as a proteosomal inhibitor.

### 2.2. Cell Culture

Human gastric adenocarcinoma cell lines MKN74 (kindly provided by Carla Oliveira, University of Porto) NCI-N87 (ATCC^®^ CRL-5822™; ATCC, Manassas, VA, USA), AGS (ATCC^®^ CRL-1739™), and AGSEcad (described in [9]) were maintained in RPMI 1640 (Gibco®, Thermo Fisher Scientific) supplemented with 10% fetal bovine serum (HyClone™, GE Healthcare Life Sciences, Logan, UT, USA) agnd 100 U-100 µg/mL penicillin G–streptomycin sulfate (Gibco^®^) at 37 °C under 5% CO_2_ humidified atmosphere. For infection experiments with *H. pylori*, gastric cell lines were grown in antibiotic-free medium at 100% confluence for 5 days in 6-well or 12-well plates (TPP^®^ Plastic Products AG, Trasadingen, Switzerland), with medium changes carried out every other day and overnight before the infection experiment. Human umbilical vein endothelial cells (HUVECs; HUV-EC-C, ATCC^®^ CRL1730™) were maintained in medium 199 (M199) with Earle’s salts, stable glutamine, and 25 mM HEPES (Biowest, Nuaillé, France) supplemented with 10% FBS (HyClone™), 100 U-100 µg/mL penicillin G–streptomycin sulfate (Gibco^®^), 100 µg/mL heparin (Sigma-Aldrich, MI, USA), and 30 µg/mL BTI endothelial mitogen (ECGS) (BioMedical Technologies Inc, Stoughton, MA, USA) in gelatin-coated (Sigma-Aldrich) tissue-culture petri dishes (TPP^®^ Techno Plastic Products AG) at 37 °C under 5% CO_2_ humidified atmosphere. All cell lines were passaged less than 10 times and were Mycoplasm-free tested by PCR using the Venor^®^ GeM Advance kit (Minerva Biolabs GmbH, Berlin, Germany). Cell lines were genotyped for 15 short tandem repeats (STRs) plus Amelogenin marker for gender identification (Promega Powerplex ^®^ 16, Promega Corp., Fitchburg, WI, USA; and AmpFLSTR Identifiler^®^, Applied Biosystems™, Beverly, MA, USA) and the results were compared with international databases to confirm the identity of the cell lines.

### 2.3. Cell Viability/Proliferation Assay

The viability of confluent monolayers in the presence and absence of *H. pylori* was estimated using the CellTiter 96^®^ AQueous One Solution Cell Proliferation Assay (Promega Corp.). Cells were plated at 100% confluence in 96-well plates in antibiotic-free medium containing 10% FBS for 5 days, after which they were infected at MOI100 for 24 h or treated with equal volume of saline solution (uninfected control). Twenty microliters of the CellTiter 96^®^ AQueous One Solution Reagent was added directly to the culture wells and incubated for 1 h. The absorbance was measured at 490 nm with a BioTek Synergy Mx 96-well plate reader (BioTek Instruments, Inc., Winooski, VT, USA).

### 2.4. H. Pylori Strains and Clinical Isolates

*H. pylori* strains 26695 (ATCC^®^ 700392™, *cagPAI*+, *vacA* s1/m1), 60190 (ATCC^®^ 49503™, *cagPAI*+, *vacA* s1/m1), 84-183 (ATCC^®^ 700392™, *cagPAI*+, *vacA* s1/m1), Tx30a (ATCC^®^ 51932™; *cagPAI*-, *vacA* s2/m2), and the insertion mutants with inactivation of the *cagA* (Δ*cagA*), *cagE* (Δ*cagE*), or *vacA* (Δ*vacA*) genes of the 60190 and 84-183 strains, kindly gifted by John Atherton, were used for the infection experiments. Four *H. pylori* clinical isolates from our lab collection were also used, namely, CI-50 (*cagA*+, *vacA* s1/m1), CI-62 (*cagA*-, *vacA* s1/m1), CI-64 (*cagA*+, *vacA* s1/m1), and CI-65 (*cagA*+, *vacA* s1/m1). Bacteria were maintained for 48 h in Trypticase™ Soy Agar with 5% sheep’s blood (TSAII; Becton, Dickinson and Company, Franklin Lakes, NJ, USA) at 37 °C under microaerophilic atmosphere (GENbox microaer; bioMérieux S.A., Marcy l’Etoile, France) and minimally passaged (maximum 10 passages). In experiments simultaneously using wild-type and mutants of the 60190 strains, bacteria were cultured on Brain Heart Infusion (BHI) agar medium (Becton Dickinson GmbH, Heidelberg, Germany) supplemented with 10% sheep’s blood (Probiológica Lda., Lisboa, Portugal) plus kanamycin (50 μg/mL; Thermo Fischer Scientific), but only for the mutants.

### 2.5. Infection of Gastric Cell Lines

*H. pylori* colonies grown on blood agar plates for 48 h were collected in phosphate buffer saline (PBS; pH7.2) and the density was estimated by spectrophotometry (Optical Density, OD, at 600 nm). Unless stated otherwise, bacterial cells were added to the monolayer of gastric epithelial cells at a multiplicity of infection (MOI) of 100 for defined time-points. Cocultures were maintained at 37 °C under 5% CO_2_ humidified atmosphere. Uninfected cultures (controls) were processed similarly, with the addition of PBS instead of the bacteria inoculum. After infection, the cell culture supernatants were collected and processed for conditioned media preparation when needed, and washed 3× with PBS solution with Ca^2+^ and Mg^2+^ (Biochrom GmbH, Berlin, Germany) for the preparation of total lysates. In experiments with the chemical inhibitors PP2, Dasatinib, and CAY10626, cells were pre-incubated for 1 h before infection; for experiments with Concanamycin A, Bafilomycin A1, and bortezomib, 1 h after incubation with inhibitors, cells were washed 3× with cell culture medium and then infected with bacteria for 24 h.

### 2.6. Immunofluorescence

Cells grown on coverslips, infected or not (control) with *H. pylori*, were washed with PBS-Ca^2+^/Mg^2+^ and fixed in 4% paraformaldehyde (Polysciences Inc., Warrington, PA, USA) in PBS (pH 7.2) for 20 min at room temperature. Subsequently, cells were permeabilized and blocked with 5% goat serum–0.3% Triton X-100 in PBS for 1 h at room temperature, followed by sequential incubations with unconjugated primary and fluorochrome-conjugated secondary antibodies for 1 h at room temperature, with several washes in PBS between incubations. Coverslips were mounted on slides with Vectashield^®^-DAPI (Vector Laboratories, Burlingame, CA, USA) and viewed with a Zeiss Axio Imager Z1 upright fluorescence microscope (Carl Zeiss, Oberkochen, Germany) or with a Leica TCS-SP5 laser scanning confocal microscope (Leica Microsystems, Wetzlar, Germany).

### 2.7. Immunoblotting

Total cell lysates from infection and transfection experiments were prepared in cold lysis buffer (1% Triton X-100, 1% NP-40 in PBS, pH 7.4) containing a cocktail of protease (Roche Applied Science, Mannheim, Germany) and phosphatase (Sigma-Aldrich) inhibitors. Protein concentration was determined by the DC protein assay (Bio-Rad Laboratories Inc., Hercules, CA, USA). Samples of 10–20 μg were diluted in 4x Laemmli buffer (Bio-Rad) with β-mercaptoethanol (Sigma-Aldrich), denaturated at 95 °C for 5 min, separated onto 10% SDS-PAGE gels, and transferred onto 0.45 µm pore-size nitrocellulose membrane (Bio-Rad). Membranes were blocked with 5% non-fat milk in Tween 0.1%–PBS or with 5% BSA in PBS, incubated with primary and horseradish peroxidase (HRP)-conjugated secondary antibodies, washed several times with TBS–0.5% Tween 20, and detected with a chemiluminescent HRP detection reagent (Luminata FORTE, Merck Millipore, Darmstadt, Germany or Clarity™ Western ECL Substrate, Bio-Rad). Bands were quantified by densitometric analysis using Quantity One® software (Bio-Rad).

### 2.8. Detection of Tyrosine-Phosphorylated EPHA2 by Enzyme-Linked Immunosorbent Assay

A sandwich ELISA based on a 3,3’,5,5’-tetramethylbenzidine (TMB) and horseradish peroxidase (HRP) system was performed to measure tyrosine-phosphorylated EPHA2 levels in total cellular lysates (200 µg of total protein) from uninfected and infected cultures using the DuoSet IC Human Phospho-EPHA2 ELISA kit (R&D Systems, Minneapolis, MN, USA), following the manufacturer’s protocol. Optical density was measured at 560 nm using a microplate reader (BioTek Instruments, Inc.).

### 2.9. RNA Extraction, cDNA Synthesis, and Quantitative RT-PCR (RT-qPCR)

Total RNA was extracted using the PureLink® RNA Mini Kit Isolation Kit (Ambion^®^, Thermo Fisher Scientific), following the manufacturer’s instructions. RNA (500 ng) was reversed-transcribed using Superscript-II-Reverse-Transcriptase and random-hexamers (Invitrogen). Quantitative PCR (qPCR) was performed in the Applied Biosystems 7500 Fast Real-Time PCR System (Applied Biosystems^®^, Waltham, MA, USA) using TaqMan Gene Expression Assays (Applied Biosystems) for the EPHA2 (Hs00171656_m1; Applied Biosystems^®^); endogenous control GAPDH (Hs99999905_m1; Applied Biosystems^®^) was used to normalize the gene expression. Data were analyzed by the comparative 2^(−ΔΔCт) method [46], using uninfected or nonsilencing cells as references.

### 2.10. Small Interfering RNA (siRNA) Transfections

Transient transfection experiments were performed using siRNA targeting EPHA2 (Hs_EPHA2_5, SI00300181; Qiagen^®^, Hilden, Germany), AllStars negative control as a nonsilencing siRNA control (#1027281; Qiagen^®^), and Lipofectamine® 2000 transfection reagent (Invitrogen™ Life technologies, CA, USA), according to the manufacturer’s instructions with slight modifications. siRNAs were used at a final concentration of 50 nM in serum- and antibiotic-free Opti-MEM medium (Invitrogen). Cells at 50% confluence (5 × 10^5^ cells) grown in 6-well plates were incubated overnight with the transfection mixture, then washed and cultured in normal growth medium without antibiotics from 1 to 3 days post-transfection, unless otherwise stated. Bacterial infection was performed at a MOI of 100 for the last 24 h. The silencing efficiency was evaluated by immunoblotting.

### 2.11. Cell–Cell Adhesion Assay

The slow aggregation assay was used to evaluate cell–cell adhesion. Briefly, 0.67% of Bacto™ Agar (Difco BD Biosciences, Sparks, MD, USA) was dissolved with sterile PBS (pH 7.4) in a microwave. Once a temperature of about 50 °C was reached, 100 μL of agar suspension was transferred to each well of a 96-well plate and allowed to solidify at 4 °C on a horizontal surface. Two-hundred microliters of complete medium containing 20,000 cells (except in NCI-N87 cells, for which 50,000 cells were used), were plated over the agar and incubated for 5 days in standard culture conditions. Measurement of the area of cell aggregates was performed using Quantity One software (BioRad).

### 2.12. Adhesion Assay to ECM Substrates

Cell adhesion assays were performed in 96-well flat-bottom microtiter plates (TPP) coated with either collagen I (Millipore, 08-115), collagen IV (Sigma, C6745), fibronectin (Biochrom GmbH, L7117), or laminin (Sigma, L4544), Vitronectin (BD Biosciences, 354238) at 5 µg/mL overnight at 4 °C. Coated wells with 5 µg/mL of poly-L-Lysine (Biochrom GmbH, L 7240) and 0.5% BSA in DPBS were used as the maximal adhesion-positive control and the minimal adhesion-negative control, respectively [47]. Prior to cell seeding, the plates were blocked for nonspecific-binding with 0.5% BSA (w/v) in DPBS (Invitrogen) containing Pen/Strep (Invitrogen) for 2 h at 37 °C. One hundred microliters of cell suspension (1 × 10^6^ cells/mL) was seeded in serum-free medium for 60 min at 37 °C in standard culture conditions. Subsequently, the plates were washed with DPBS to remove nonadherent cells, then fixed with acetone:methanol (1:1) for 10 min at 4 °C, except for poly-L-lysine-coated wells, which were fixed in 4% paraformaldehyde. The absorbance was measured at 570 nm with a microplate reader. The attachment of cells to wells coated with poly-L-Lys (Biochrom GmbH) was defined as 100% of adhesion.

### 2.13. In Vitro Matrigel Invasion Assay

Matrigel-coated 24-well invasion inserts of 8-μm pore filters (Corning™ 354480, BD Biosciences, Bedford, MA, USA) were used for the in vitro invasion assay according to the manufacturer’s instructions, with some modifications. Briefly, after filter rehydration with antibiotic-free medium supplemented with 10% FBS in both chambers, 5 × 10^4^ cells were transferred into the Transwell and incubated for 24 h at 37 °C in the presence or absence of *H. pylori*. After this period of incubation, the filters were washed and noninvasive cells inside the Transwell (at upper side of the membrane) were removed with a wet cotton swab. Invasive cells (at the lower side of the membrane) were fixed in 4% paraformaldehyde, mounted with Vectashield^®^ with DAPI (Vector Laboratories), and scored in the whole filter using 20× magnification.

### 2.14. In Vitro Angiogenesis Assay—Endothelial Cell Capillary-Like Tube Formation Assay

HUVECs (6 × 10^4^) cells were seeded in 96-well plates coated with growth factor-reduced Matrigel™ (Corning^®^ Inc., Bedford, MA, USA) in the presence of conditioned media from MKN74 cells, which were transiently silenced with siEPHA2, nonsilenced, or treated with lipofectamine, and allowed to stabilize for 3 h in a cell culture incubator at 37 °C with 5% CO_2_ humidified atmosphere. Endothelial-like network formation was followed in the center of each well using a Leica DMI 6000 time-lapse microscope (Leica Microsystems, Wetzlar, Germany) for 2 h, with 10× magnification and z-stacks of 2.08 µm acquired every 30 min. The number of tubes and branching points per microscopic field were automatically quantified using Ibidi Quantitative Tube Formation Image Analysis—WimTube software (Onimagin Technologies SCA, Córdoba, Spain).

### 2.15. In Vivo Angiogenesis Assay—Chicken Embryo Chorioallantoic Membrane (CAM)

The chicken embryo chorioallantoic membrane (CAM) model was used to evaluate the in vivo angiogenic potential [48] of siEPHA2-transfected MKN74 cells in comparison with that of parental MKN74 (treated with lipofectamine (lipo)) and nonsilencing transfected MKN74 (siNS) (n = 15 fertilized eggs for each experimental group) cells. Fertilized chick (*Gallus gallus*) eggs were incubated horizontally at 37.5 °C in a humidified atmosphere and referred to embryonic development day 0 (E0). After 3 days (E3), 2 mL of albumen was withdrawn and a square window was opened in the eggshell. The window was sealed with adhesive tape and the eggs returned to the incubator until E10. At E10, 1 × 10^6^ MKN74 cells per embryo were resuspended in 10 µL of antibiotic-free and serum-free medium and were placed on top of the CAM within a 5 mm silicon ring under sterile conditions. The eggs were resealed and returned to the incubator for an additional 3 days until they reached the E13 stage. The embryos were euthanized by adding 2 mL of 10% neutral-buffered formalin in the top of the CAM. After removing the ring, the fixed CAM was excised and photographed *ex ovo* under a stereoscope at 20× magnification (Olympus SZX16 coupled with a DP71 camera; Olympus Corp., Tokyo, Japan). The number of new blood vessels (smaller than 20 µm in diameter) growing radially toward the ring area was counted blind to the experimental setting.

### 2.16. Immunohistochemistry Analysis of CAM

Paraffin-embedded sections of excised formalin-fixed CAMs were deparaffinized in Clear-Rite™ 3 (Thermo Scientific™ Richard-Allan Scientific™), rehydrated through a graded ethanol series (100%, 95%, 70% ethanol), and rinsed in water. Heat-induced antigen retrieval was performed with 1× Target Retrieval Solution and citrate (pH 6.1) (DAKO, Glostrup, Denmark) for 35 min. Endogenous peroxidase activity was blocked for 5 min with DAKO Peroxidase Block reagent. After blocking, slides were sequentially incubated with a primary antibody rabbit anti-human EPHA2 (sc-924, Santa Cruz Biotechnology) and a secondary antibody with horseradish peroxidase polymer (DAKO EnVision™+ System, HRP) for 30 minutes at room temperature for each incubation. Staining was detected by incubation for 5 min with 3,3’-diaminobenzidine (DAB) (DAKO) substrate-chromogen. Counterstaining was performed with HIGHDEF® hematoxylin (Enzo Life Sciences, Farmingdale, NY, USA).

### 2.17. Angiogenesis Array

The human angiogenesis array (Proteome Profiler™ Array; R&D Systems.) was used to assess the relative expression of 55 angiogenic-related proteins (array map provided in Figure S1b) in cellular extracts of siNS- and siEPHA2-transfected MKN74 cells and in siNS-transfected MKN74 cells infected for 24 h with *H. pylori* 60190, which was used as a reference for the angiogenic response. The array membranes were probed with combined cellular extracts from 3 independent experiments with a total protein content of 250 µg per condition, according to the manufacturer’s instructions. Enhanced chemiluminescence was used to detect protein binding to the antibody array, followed by exposure to an X-ray film. The signal intensity of each antigen-specific antibody spot was quantified using Quantity One^®^ image analysis software (BioRad). For comparison of the relative expression of proteins between siNS- and siEPHA2-transfected cells in uninfected (U) and *H. pylori*-infected (I) conditions across the different arrays, the mean pixel density of the duplicated spots for each protein after subtraction of the mean pixel density of the negative control spots of the respective array was normalized for the mean pixel density of the positive control spots on the reference array (siNS_U), according to the following formula: Normalized signal intensity for protein X in array A = Mean signal density for protein X in array A * (mean signal density of positive control spots on reference array/mean signal density of positive control spots on array A). Heat map analysis using the normalized data was performed in the R software [49] using the “gplots” package. IL-8 quantification by ELISA in conditioned media from 6 independent experiments was used as a validation hit of the antibody array.

### 2.18. Quantification of IL-8 Secretion by Enzyme-Linked Immunosorbent Assay

The amount of IL-8 secreted into the cell culture medium of MKN74 gastric cells transfected with either lipofectamine, nonsilencing siRNA control, or siEPHA2 (either uninfected or infected with *H. pylori*)*,* was determined using the LEGEND MAX™ Human IL-8 ELISA Kit (BioLegend^®^, San Diego, CA, USA), according to the manufacturer’s instructions.

### 2.19. Statistical Analysis

Statistical analysis was performed using GraphPad Prism 8.1.1 software (San Diego, CA, USA). Generally, for multiple comparisons a one-way analysis of variance (ANOVA) was performed followed by a post-hoc test for pairwise comparison. For the comparison of two groups, unpaired Student’s t-test was used. The normality of the distribution was assessed by the Shapiro–Wilk test. Statistical significance was set at *p* ≤ 0.05 (**** *p* ≤ 0.0001, *** *p* ≤ 0.001, ** *p* ≤ 0.01, * *p* ≤ 0.05). Data in the graphs represented the average ± standard error (SE) of the mean of at least three experiments, unless otherwise stated.

## 3. Results

### 3.1. Infection of Polarized Gastric Epithelial Cells by H. pylori Induces Downregulation of EPHA2 Protein Independently of T4SS, CagA, and VacA Virulence Factors

**Figure 1:**
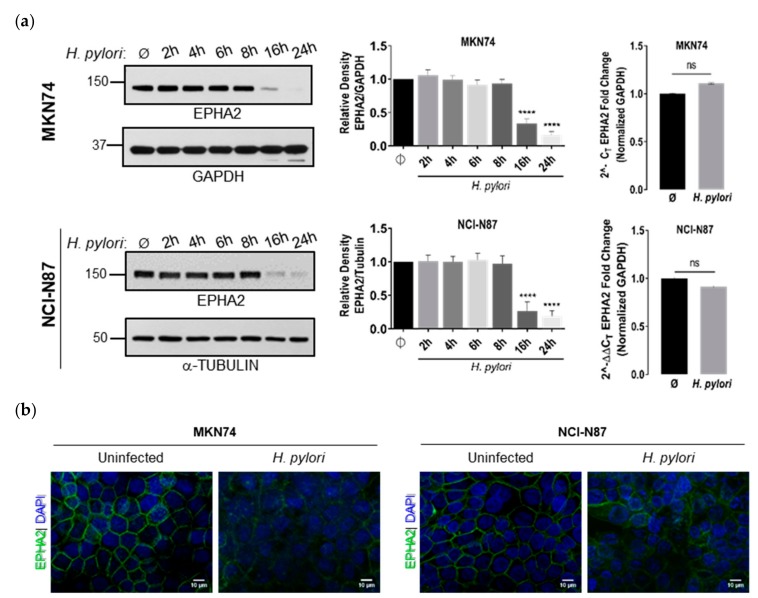

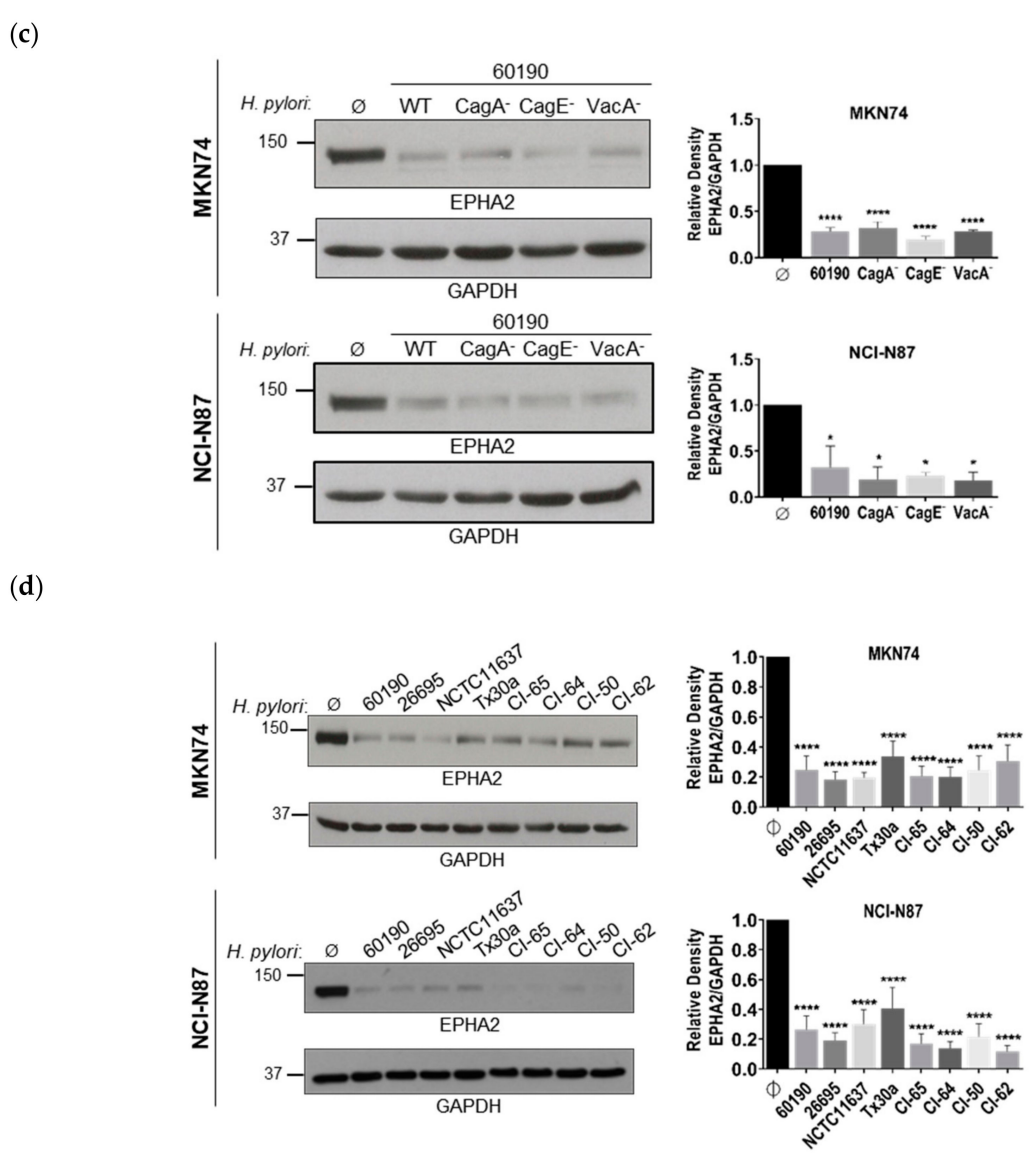
Prolonged exposure of gastric epithelial cell lines to *H. pylori* infection induced downregulation of EPHA2 receptor protein without affecting mRNA levels independently of the major virulence factors T4SS, CagA, and VacA. (**a**) EPHA2 protein expression in MKN74 (*n* = 5) and NCI-N87 (*n* = 4) cell lines either uninfected (Ø) or infected with *H. pylori* 60190 for different periods of time at an MOI of 100 assessed by Western blotting with corresponding densitometric analysis (left and middle panels); EPHA2 mRNA levels assessed by RT-qPCR at 24 h post-infection (*n* = 4; right panel); **** *p* < 0.0001; ns—not significant (*p* > 0.05). (**b**) Immunofluorescence of EPHA2 (green) protein in MKN24 cells at 24 h post-infection, with nuclei stained with DAPI (blue) (scale bar: 10 µm; 63× original magnification; *n* = 3). (**c** and **d**) EPHA2 protein expression upon coculture with (**c**) wild-type or mutants (*cagE* negative, *cagA* negative, *vacA* negative) of the H*. pylori* 60190 strain (*n* = 5 for MKN74 and *n* = 2 for NCI-N87) and (**d**) with *H. pylori* reference strains (60190, 26695, NCTC11637, Tx30a) and clinical isolates (CI-65, CI-64, CI-50, CI-62) (n = 4 for each cell line) in 24 h cocultures at MOI100 assessed by immunoblotting; **** *p* < 0.0001; * *p* ≤ 0.05. The represented densitometric analysis are the mean ± SE of all independent experiments. One-way ANOVA with post-hoc Dunnett’s test for multiple comparisons and Student’s t-test for single comparisons.

To investigate whether *H. pylori* could target the EPHA2 receptor, we performed a time-course coculture experiment with two different gastric epithelial tumor cell lines, MKN74 and NCI-N87, which were grown as monolayers for five days in confluence. Infection of both cell lines with *H. pylori* strains 60190 or 26695 induced downregulation of EPHA2 protein levels for long periods of time post-infection, as evaluated by Western blot (Figure 1a, left and middle; Figure S1), which was confirmed by immunofluorescence (Figure 1b; Figure S1). To assess if the decrease in EPHA2 protein expression upon infection was due to transcriptional regulation, we determined the relative expression of EPHA2 mRNA levels by real time quantitative PCR (RT-qPCR). No significant differences in EPHA2 mRNA levels were found between uninfected and *H. pylori*-infected cell lines 24 h post-infection (MKN74, *p* = 0.6069; NCI-N87, *p* = 0.1250; Figure 1a, right upper and bottom graphs). These results show that EPHA2 downregulation induced by *H. pylori* does not involve alterations in mRNA levels and occurs at the post-transcriptional level. No alterations in cell viability were observed 24 h post-infection in any of the cell lines, as measured by the MTS assay (Figure S1).

Next, to assess the role of the major *H. pylori* virulence factors in EPHA2 downregulation, MKN74 and NCI-N87 cell lines were infected with *H. pylori* 60190 wild-type and mutants for T4SS (type four secretion system;Δ*cagE*), CagA (Δ*cagA*), and VacA (Δ*vacA*). All mutants and the wild-type strain were equally efficient in inducing EPHA2 downregulation 24 h post-infection, demonstrating that *H. pylori*-induced EPHA2 receptor downregulation is independent of these factors (Figure 1c). Furthermore, infection of MKN74 cells with various *H. pylori* reference strains and *H. pylori* clinical isolates expressing different *cagA* and *vacA* genotypes also induced EPHA2 downregulation 24 h post-infection, showing that this effect is not specific of laboratory-modified strains, thereby corroborating the independence of EPHA2 downregulation from these virulence factors (Figure 1d).

### 3.2. H. pylori-Mediated EPHA2 Downregulation is Preceded by Activation of the EPHA2 Receptor by Phosphorylation and Followed by Protein Degradation via the Lysosomal Pathway

**Figure 2:**
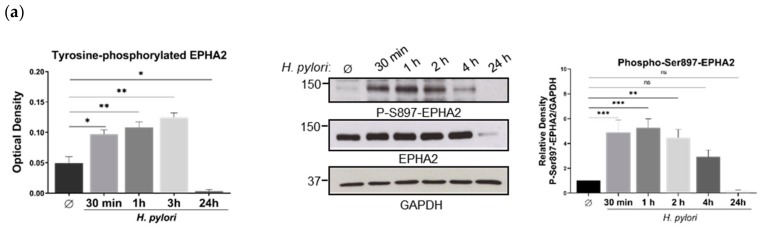

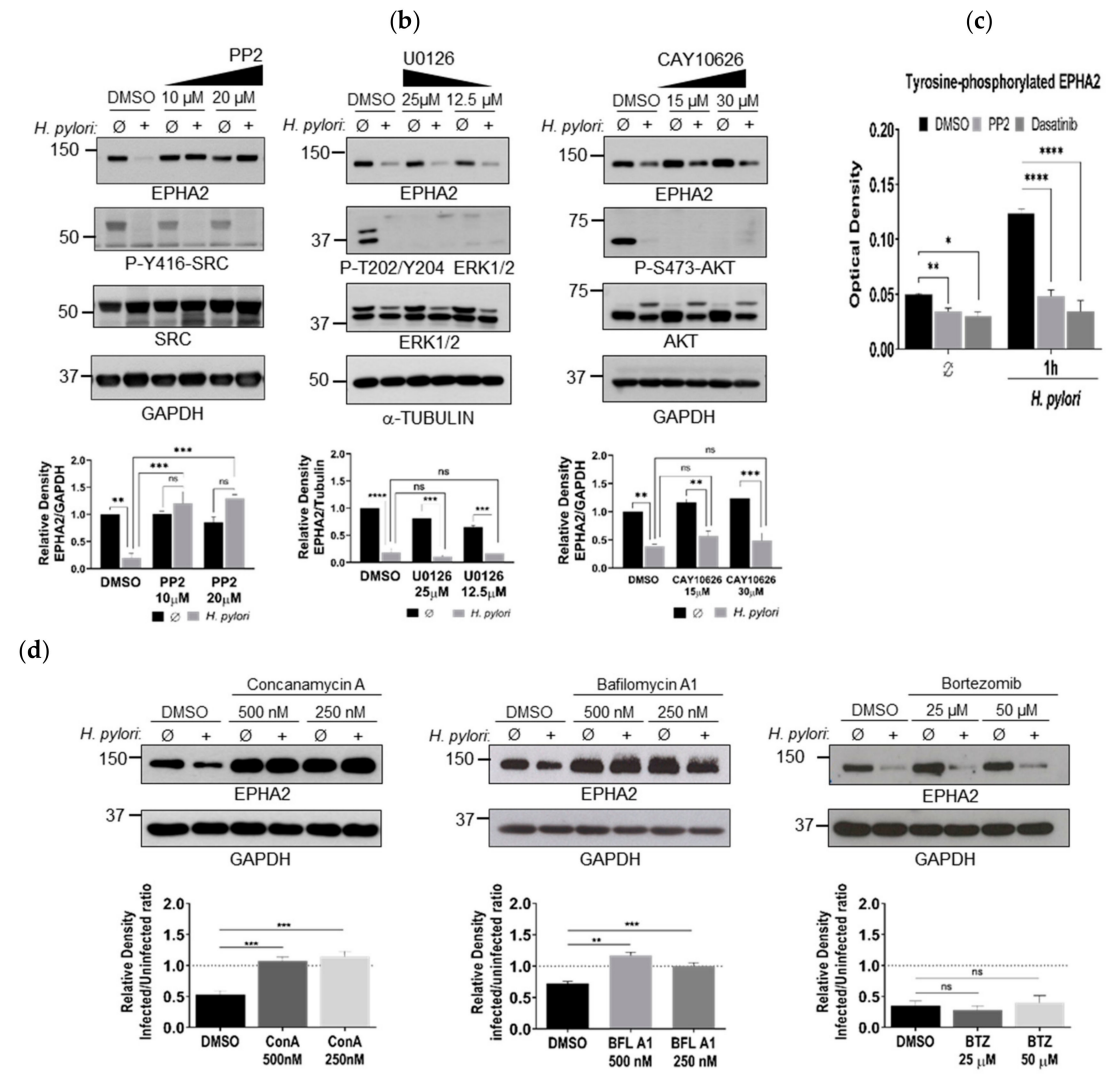
EPHA2 receptor downregulation induced by *H. pylori* infection is preceded by receptor phosphorylation early on and is followed by lysosomal degradation in the MKN74 gastric cell line. (**a**) Tyrosine and serine897 phosphorylation of EPHA2 upon *H. pylori* exposure, as determined by ELISA and Western blot, respectively; *** *p* < 0.001, ** *p* < 0.01, * *p* ≤ 0.05, ns—not significant (*p* > 0.05). (**b**) Effect of PP2 (SRC family kinase inhibitor), U0126 (MEK inhibitor), and CAY10626 (PI3Kα/mTOR inhibitor) on downregulation of EPHA2 mediated by *H. pylori* at 24 h by Western blot and corresponding quantifications by densitometry; **** *p* < 0.0001, *** *p* < 0.001, ** *p* < 0.01, ns—not significant (*p* > 0.05). (**c**) Effect of SRC family kinase inhibitors (PP2 and Dasatinib inhibitors) on EPHA2-tyrosine phosphorylation 1 h after *H. pylori* infection, as evaluated by ELISA; **** *p* < 0.0001, ** *p* < 0.01, * *p* ≤ 0.05. (**d**) Effect of lysosomal (Bafilomycin A1 and Concanamycin A) and proteasomal (bortezomib) inhibitors on EPHA2 receptor downregulation induced by *H. pylori* at 24 h post-infection as shown by Western blot and the respective relative density expressed as the ratio of infected/uninfected cells; *** *p* < 0.001; ** *p* < 0.01; ns—not significant (*p* > 0.05). One-way ANOVA with post-hoc Dunnett’s or Tukey’s test.

Next, we assessed whether downregulation of the EPHA2-induced by *H. pylori* could be due to EPHA2 activation via tyrosine phosphorylation, thereby somehow mimicking the ligand-binding activation described for EPHA2 [32,50,51] and for RTKs in general [52], leading to receptor downregulation. MKN74 cells were infected with *H. pylori* 60190 for different periods of time, and tyrosine-phosphorylation of the EPHA2 receptor was evaluated using a phospho-tyrosine EPHA2 ELISA assay. As observed in Figure 2a, EPHA2 was tyrosine-phosphorylated as early as 30 min and maintained for up to 3 h post-infection, at which point no alterations in the total protein levels of EPHA2 were observed. At 24 h post-infection, neither tyrosine-phosphorylated nor total EPHA2 were detected, suggesting receptor degradation upon activation. Since the EPHA2 receptor can also be non-canonically activated by phosphorylation at the serine897 residue [53,54], we tested whether *H. pylori* infection could induce EPHA2-S897-phosphorylation, which was observed between 30 min and until 2 h post-infection (Figure 2a).

To gain insight into which signaling pathways were involved in EPHA2 activation induced by *H. pylori* leading to receptor downregulation at late time-points (24 h), we incubated MKN74 cells with chemical inhibitors of common signaling pathways involved in EPH and EFN signaling prior to infection [25]. Using EPHA2 downregulation at 24 h post-infection as a readout of early receptor activation, we found that pre-incubation with PP2, an SRC family kinase (SFK) inhibitor, prevented *H. pylori*-induced EPHA2 downregulation when compared with vehicle-treated cells (Figure 2b). This effect was not observed when the MEK inhibitor U0216 or the PI3K inhibitor CAY10626 were used (Figure 2b). Using a phosphotyrosine ELISA for EPHA2, we found that PP2 and Dasatinib inhibitors significantly decreased tyrosine-phosphorylation of EPHA2 at 1 h post-infection (2.6×- and 3.6×-reduction for PP2 and Dasatinib, respectively; *p* < 0.0001) (Figure 2c). These results show that tyrosine-phosphorylation of EPHA2 receptor induced by *H. pylori* and its later downregulation are mediated by SFKs.

To evaluate if *H. pylori*-mediated activation of the EPHA2 receptor leads to receptor degradation like the ligand-activated RTKs, MKN74 cells were incubated with either lysosomal or proteasomal inhibitors prior to infection (Figure 2d). Pretreatment of cells with two lysosomal inhibitors, Bafilomycin A1 and Concanamycin A, significantly impaired *H. pylori*-mediated EPHA2 downregulation. This effect was not detected with the proteasomal inhibitor bortezomib. These results show that the lysosomal, but not the proteasomal, pathway contribute to the degradation of EPHA2 receptor upon activation by *H. pylori* infection as the mechanism of post-transcriptional regulation of EPHA2 downregulation, similarly to ligand-activated RTKs.

Altogether, these results establish that *H. pylori* infection activates the EPHA2 receptor in MKN74 gastric cells via phosphorylation at early time-points after infection, ultimately leading to receptor degradation for longer periods post-infection. *H. pylori*-mediated activation of EPHA2 induces both tyrosine and serine897 phosphorylation, mimicking ligand-dependent activation associated with tyrosine-phosphorylation of the receptor and ligand-independent mechanisms, which are linked to the phosphorylation of the serine897 residue of the EPHA2 receptor.

### 3.3. EPHA2-Silencing by siRNA on Gastric MKN74 and NCI-N87 Cell Lines Impairs Cell–Cell and Cell–Matrix Adhesion and Favours Cell Invasion 

**Figure 3:**
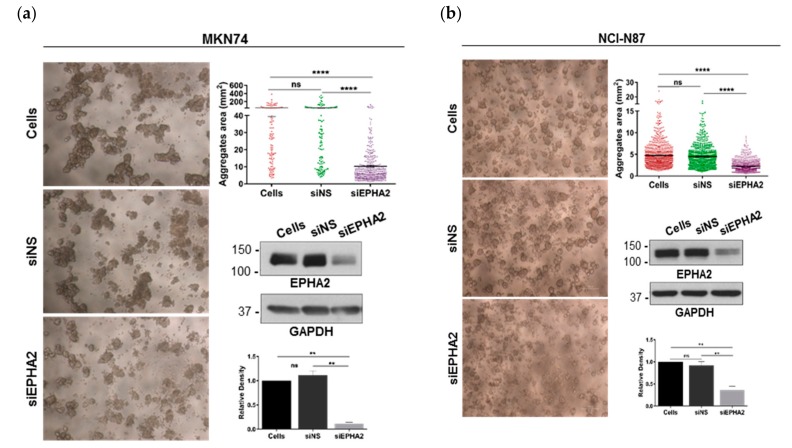

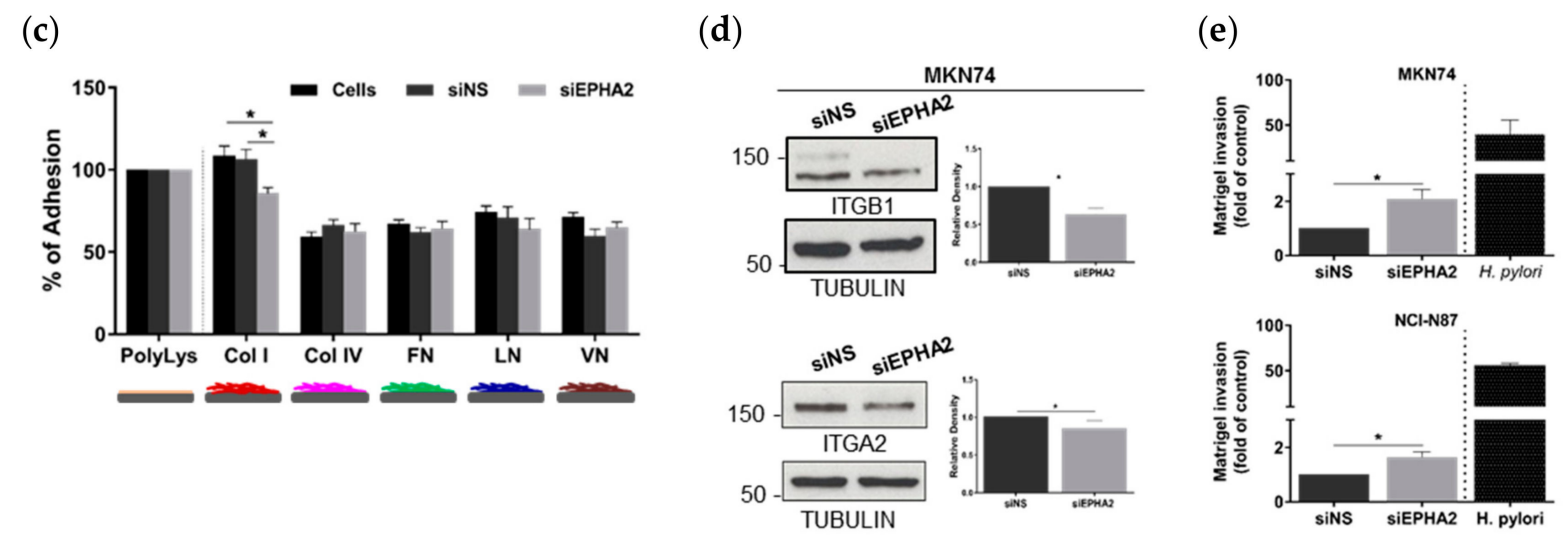
Role of EPHA2 in cell–cell and cell–matrix adhesion and invasion of gastric cells. Representative images of the slow aggregation assay for (**a**) MKN74 and (**b**) NCI-N87 gastric cells untreated (cells) transfected with a nonsilencing siRNA (siNS) or with a EPHA2 siRNA (siEPHA2); **** *p* < 0.0001, ** *p* < 0.01, ns—not significant (*p* > 0.05). Cell–cell aggregate size quantification for each condition using Quantity One software. Representative Western blot of the EPHA2 protein expression and the densitometric quantification of the EPHA2 levels in all slow aggregation assays (*n* = 4). (**c**) Cell–matrix adhesion of nontransfected siNS or siEPHA2-transfected MKN74 cells to different substrates using poly-L-lysine-coated cells as the maximal adhesion (100%); * *p* ≤ 0.05. (**d**) Protein expression of the integrins beta 1 (ITGB1) and alpha2 (ITGA2), a major receptor for collagen type I, assessed by Western blot and respective densitometric quantification (*n* = 2); * *p* ≤ 0.05. (**e**) *In vitro* Matrigel invasion assay of MKN74 and NCI-N87 cells either transfected with siNS or siEPHA2 upon infection with *H. pylori* 26695 strain as a control; * *p* ≤ 0.05. Student’s t-test and one-way ANOVA with post-hoc Tukey’s test.

To mimic the effects of *H. pylori* infection on EPHA2 downregulation and to unravel the functional effects of EPHA2 in gastric cell–cell adhesion, cell–matrix adhesion, and invasion, we transiently transfected gastric cells with an siRNA targeting EPHA2 mRNA (siEPHA2) or a nonsilencing siRNA control (siNS). Successful knockdown of EPHA2 was confirmed by immunoblotting. First, the function of EPHA2 on cell–cell adhesion was assessed by the slow aggregation assay. EPHA2-silenced MKN74 (Figure 3a) and NCI-N87 (Figure 3b) cells formed significantly smaller aggregates than untransfected and siNS-transfected cells, thereby supporting a role for EPHA2 in the establishment of cell–cell adhesion between gastric cells. Next, we assessed the effect of EPHA2 knockdown on the adhesion of gastric cells to different extracellular matrix components. These included structural substrates, such as type I and IV collagens, fibronectin, and laminin, or extracellular matrix substrates involved in tissue remodeling, such as vitronectin (Figure 3c). Cells lacking EPHA2 adhered significantly less to collagen type I-coated surfaces than untreated and siNS-transfected cells. No significant differences were found regarding the adhesion of gastric cells to collagen type IV, fibronectin, laminin, or vitronectin. After observing that EPHA2 knockdown affected cell–matrix adhesion to type I collagen, we tested via immunoblotting the expression of the α2β1 integrin receptor, a functional cellular receptor for type I collagen [55]. MKN74 cells without EPHA2 expressed significantly less total α2 and β1 integrin receptors compared with control cells treated with siNS (Figure 3d), showing that cell adhesion to type I collagen is mediated by the EPHA2–integrin axis. Subsequently, since EPHA2 regulates both gastric cell–cell and cell–matrix adhesion, we tested its involvement on cell invasion using the in vitro Matrigel invasion assay. EPHA2 silencing in both MKN74 and NCI-N87 cells increased cell invasion by approximately 2-fold in comparison with the respective siNS-transfected control cells, although to a lesser extent than invasion induced by the bacteria, which involves several signaling pathways simultaneously (Figure 3e). Altogether, these results show that in the gastric cell context, EPHA2 promotes cell–cell and cell–matrix adhesion and suppresses cell invasion, processes that are likely affected by the targeting of EPHA2 by *H. pylori*.

### 3.4. EPHA2 Receptor Influences the Angiogenic Expression Profile of Gastric Epithelial Cells

**Figure 4:**
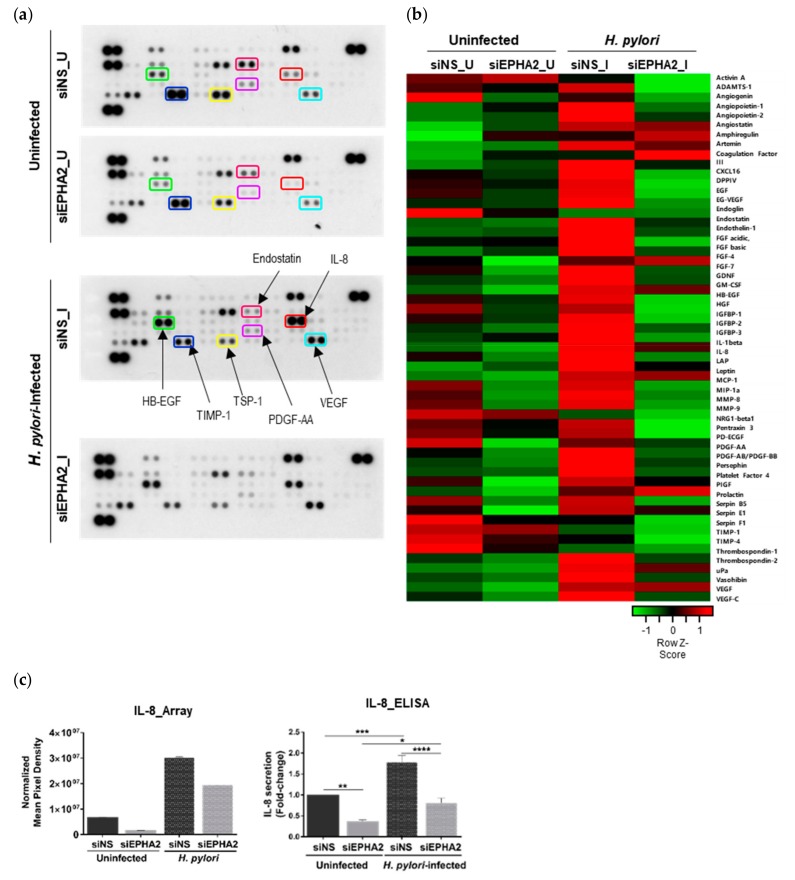
Angiogenic signature imprinted by EPHA2 in gastric cells. (**a**) A human angiogenesis antibody array composed by duplicated spots of 55 angiogenic-related factors was performed with a pool of cell lysates from siNS and siEPHA2-transfected MKN74 and siNS cells-infected with *H. pylori* 60190 strain (24 h; MOI100) as a control of the angiogenic response (*n* = 1); some representative angiogenic factors were highlighted. The map of the array and graph with fold-change variations are presented in Supplementary Figure S1c,d (**b**) Heat map analysis representing the siNS-normalized average pixel density of the duplicated spots for each angiogenic-related protein in the array. (**c**) Validation of the array for IL-8 by ELISA (*n* = 6) and its comparison with array expression. One-way ANOVA with post-hoc Tukey’s test for multiple comparisons analysis: **** *p* < 0.0001; *** *p* < 0.001; ** *p* < 0.01 * *p* ≤ 0.05; ns—not significant (*p* > 0.05).

Given the importance of the EPH receptors for vasculogenesis and angiogenesis [21,22,28], we determined the impact of *H. pylori*–EPHA2 interaction on angiogenesis. To obtain a global overview of the EPHA2-regulated angiogenic factors, we used an antibody angiogenesis array. The Human Angiogenesis Proteome Profiler Array was probed with a pool of cellular extracts of uninfected and *H. pylori*-infected MKN74 cells, which were transfected with either siNS or siEPHA2 (Figure 4a). The array analysis (Figure 4b, Figure S1c, and Table S1) clearly shows that *H. pylori* triggers a strong angiogenic response in MKN74 gastric cells, and that the EPHA2 receptor participates, either directly or indirectly, in the angiogenic process by regulating angiogenic factors. *H. pylori* infection (siNS_I) induces the expression of numerous pro-angiogenic factors with a fold-change of ≥1.5 when compared to the siNS_U control (Table 1).

Interestingly, EPHA2-silencing in the context of infection alters the expression of some angiogenic-related factors to levels that are lower (fold-change of ≤0.5) or higher (fold-change of ≥1.5) than those of siEPHA2 silencing in the uninfected setting (siEPHA2_U), thereby pointing to its involvement in crosstalk with other angiogenic-regulating genes triggered by *H. pylori* infection (Table 2).

In uninfected conditions, the silencing of EPHA2 (siEPHA2_U) directly affects the expression of GDNF, CXCL8/IL8, CCL3/MIP-1alpha, MMP9 (matrix metalloproteinase 9), and PDGF-AA (platelet derived growth factor subunit A), with fold-changes of ≤0.5 when compared with siNS_U. CXCL8/ IL-8 was selected as a representative hit for the validation of the array, since it is one of the most significantly upregulated genes during *H. pylori* infection [57]. The quantification of IL-8 levels by ELISA confirmed the array data showing that IL-8 protein expression is partially regulated by the EPHA2 receptor in both uninfected and *H. pylori*-infected conditions in MKN74 gastric cells (Figure 4-c).

Overall, the results obtained using the antibody angiogenesis array highlight the contribution of EPHA2 for the regulation of several angiogenic-factors in response to *H. pylori*-infection, thereby mimicking the situation of EPHA2 knockdown. 

### 3.5. Knockdown of EPHA2 Receptor in Gastric Cells Impairs Angiogenesis In Vivo and In Vitro 

**Figure 5:**
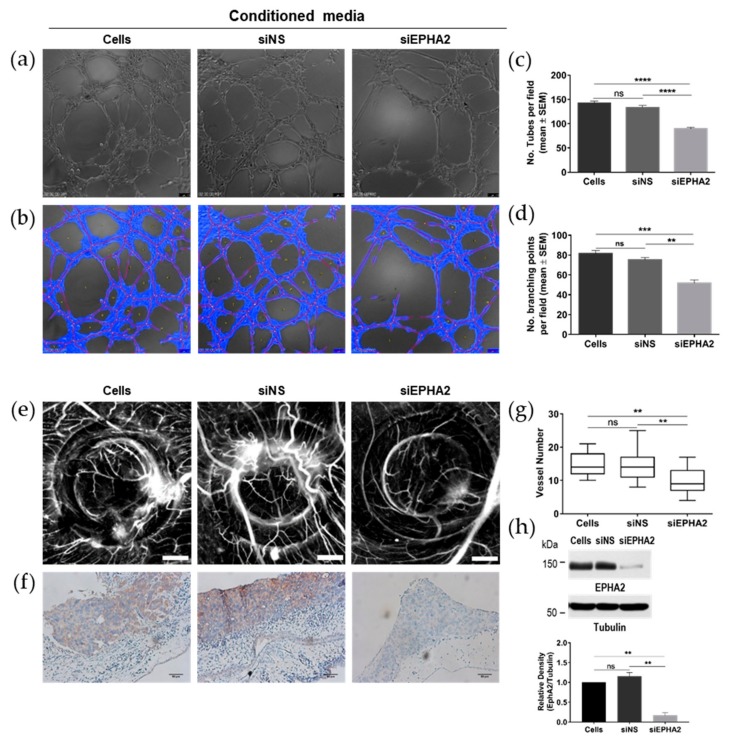
Role of EPHA2 in angiogenesis *in vitro* (**a**–**d**) and *in vivo* (**e**–**h**). (**a**) Representative micrographs of the *in vitro* capillary-like structures formed by human umbilical vein endothelial cells (HUVECs), upon treatment with conditioned medium from untreated MKN74 cells (cells), MKN74 cells treated with a nonsilencing siRNA (siNS) as a negative control, or with an siRNA for the EPHA2 (siEPHA2) 5 h post-seeding in Matrigel-coated wells. (**b**) Corresponding automatic analysis using WimTube software (scale bar: 100 µm; original magnification: ×100) with the quantification of the number of tubes (**c**) and branching points (**d**) per microscopic field from 3 independent experiments. (**e**) Representative photomicrographs of the *in vivo* chicken embryo chorioallantoic membrane (CAM), depicting new blood vessel formation induced by untreated MKN74 cells (cells), MKN74 cells transfected with a nonsilencing siRNA negative control (siNS), or with an siRNA against the EPHA2 (siEPHA2). Cells were inoculated on top of the CAM inside a 5 mm silicon ring under sterile conditions for 3 days (scale bar: 1 mm; original magnification: 20×). (**f**) Representative immunohistochemistry of the CAM paraffin sections stained with EPHA2 antibody (scale bar: 50 µm; original magnification: 200×). (**g**) Quantification of the number of new vessels radially formed toward the inoculation area as a measure of the angiogenic potential of the inoculated cells. Data regarding 15 fertilized eggs per condition are depicted on the box plot graph. (**h**) Representative Western blot of EPHA2 and tubulin expression in MKN74 for the different experimental conditions at the end of the experiment, and the quantification for 3 independent experiments. Data are presented as mean ± SE. One-way ANOVA analysis followed by Tukey’s multi-comparison test: **** *p* < 0.0001; *** *p* < 0.001; ** *p* < 0.01; ns—not significant (*p* > 0.05).

Following the observation that EPHA2 receptor regulates the production of several angiogenic-related proteins in gastric MKN74 cells, we used two angiogenesis assays to assess its functional effect. First, to understand if the angiogenic factors regulated by EPHA2 in gastric cells favour angiogenesis, we used conditioned media collected from untreated (cells), siNS-treated, and siEPHA2-treated MKN74 cells and tested them *in vitro* on human umbilical vein endothelial cells (HUVECs) for the induction of tube-like structures (Figure 5a–d). The tube-like structures started to form 2 h after the seeding of HUVECs onto Matrigel with conditioned media and stabilized at 5 h post-plating. Conditioned media from siEPHA2-treated MKN74 cells significantly impaired tube formation when compared with conditioned media from untreated MKN74 cells and siNS-MKN74 cells (Figure 5a,b), which was assessed by the quantification of the total number of tubes (Figure 5c) and the total number of branching points (Figure 5d) per microscope field. Similar results were obtained for the NCI-N87 cell line (Figure S1e).

These results demonstrate that the secreted angiogenic factors directly regulated by the EPHA2 receptor overall confer a pro-angiogenic potential to gastric cells.

Next, we performed the chick embryo chorioallantoic membrane (CAM) *in vivo* angiogenesis assay using the same set of cells, i.e., untreated (cells), siNS-treated, and siEPHA2-transfected cells, and inoculated them in the CAM (Figure 5e). The angiogenic response toward these inoculated MKN74 cells was quantified by counting the number of novel radial blood vessels that formed. EPHA2-silenced MKN74 cells elicited the formation of significantly fewer blood vessels than the untreated and siNS-transfected MKN74 cells (Figure 5g), showing that EPHA2 expression on gastric cells contributes to their angiogenic potential *in vivo*. The CAM-inoculated MKN74 cells were at day 0 of transfection; silencing efficiency was confirmed by immunohistochemistry for the EPHA2 in CAM sections collected at the end of the CAM assay (Figure 5f) and by immunoblotting with cell lysates from cells at 3 days post-transfection (Figure 5h).

Together, data from both the *in vitro* and *in vivo* angiogenesis assays show that EPHA2 is a key regulator in the angiogenesis process promoted by gastric epithelial tumor cells, reinforcing the functional importance of the targeting of this receptor by *H. pylori*. The mechanism involves induction and/or secretion of angiogenic-related proteins, as well as ligand interactions between tumor cells and the endothelium.

## 4. Discussion and Conclusions

Manipulation of host signaling pathways by pathogens is an important strategy for their successful survival and persistence with collateral consequences linked to the development of pathogen-associated diseases [58,59]. In the case of *H. pylori*, among the multiple signaling pathways that can be co-opted by the infection, those associated with RTKs have lacked entire exploration up until now.

This study uncovered the molecular mechanisms underlying EPHA2 RTK deregulation in the presence of *H. pylori* infection and its role in manipulating key host cellular functions. Using an in vitro infection culture system, in which gastric epithelial cancer cells were left in confluence for five days to establish a polarized monolayer mirroring epithelial cell–cell interactions, we observed that *H. pylori* induced EPHA2 downregulation at 16 hours post-infection without affecting mRNA levels, independently of the major virulence factors T4SS, CagA, and VacA. Pre-treatment of cells with chemical inhibitors demonstrated that EPHA2 downregulation was mediated by SRC family kinases and occurred via the lysosomal degradation pathway. Further, EPHA2 degradation induced by longer periods of exposure to *H. pylori* was preceded by receptor activation through phosphorylation (tyrosine and serine897) as early as 30 minutes post-infection in the absence of growth factors. Although we did not further dissect which tyrosine residues were phosphorylated, a phosphoproteomic analysis performed by Glowinski and colleagues using the AGS gastric cancer cell line detected EPHA2 tyrosine-phosphorylation at Y575 and Y588/Y594 residues of the juxtamembrane region upon infection with the *H. pylori* P12 strain using SILAC-LC-MS and phospho-tyrosine antibody enrichment [45]. All of the P12 strains tested (i.e., wild-type, deltaCagA, and deltaPAI) were able to induce EPHA2 tyrosine-phosphorylation at the Y588/Y594 residue 90 minutes post-infection, although at different levels, whereas phosphorylation at the Y575 residue was only detected 7 hours after infection in the wild-type P12 strain [45]. In the canonical model of RTK activation, ligand binding induces receptor activation via phosphorylation of key tyrosine residues at the kinase domain of the receptor leading to rapid internalization via endocytosis and subsequently sorting of internalized ligand-RTK complexes to lysosomes for degradation as a termination signal of receptor activation [52]. Nevertheless, increasing evidence points toward a signaling role of several RTKs within the endocytic compartment after internalization, as well as to its recycling back to the cell surface from peripheral endosomes or recycling endosomes, eventually resulting in sustained signaling [60,61]. Boissier et al. showed that activated EPHA2 is degraded in the lysosomes following ligand-mediated activation and that about 35% of internalized receptors are recycled back to the plasma membrane, demonstrating that EPHA2 retains the capacity to signal in endosomes [62]. Should these mechanisms apply to EPHA2-targeting by *H. pylori*, important functional consequences could occur.

Notable, the effects of *H. pylori* infection on EPHA2 receptors, in particular the activation by tyrosine and serine phosphorylation and receptor downregulation, match the ligand-dependent and -independent pathways for EPHA2 activation, respectively, described in several tumor-derived cellular models. Stimulation of EPHA2 receptor with soluble ephrin A1-Fc (the highest affinity ligand for EPHA2) induces EPHA2 receptor activation by tyrosine phosphorylation, leading to receptor internalization and downregulation [32,50,51]. This ligand-mediated EPHA2 activation in cancer cells has conflicting outcomes regarding tumor adhesion, migration, invasion, and angiogenesis, which have been attributed to distinct cellular and receptor types in a context-dependent manner [25,54,63,64,65,66]. Another layer of complexity in EPH receptor activation with functional consequences is the clustering state of ephrin ligands and of EPH receptors [30,67,68,69,70,71]. The oligomerization form in which the ephrin-A1 ligand is presented to an EPHA2 receptor determine if EPHA2 is activated or not, and which signaling pathways are triggered further contributing to the diverse functional activities of the EPHA2 receptor [72,73]. In addition to the canonical activation common to RTKs, EPHA2 can also be activated in a ligand-independent (noncanonical) manner related to S897-phosphorylation and associated withcancer cell motility, invasion, and progression [53,54].

Despite our finding that *H. pylori* infection induces both tyrosine and serine phosphorylation of the EPHA2 receptor early on after infection preceding receptor downregulation, we cannot rule out the possibility that EPHA2 activation induced by *H. pylori* may involve crosstalk with other RTKs, which is characteristic of EPH receptors [24,74,75].Particularly RTKs expressed in gastric epithelial cells and reported to be activated by *H. pylori*, such as c-MET/HGF receptor and EGFR. Future studies are required to address which phospho-residues are bacteria-modified and to disentangle which bacterial components are responsible for EPHA2 activation that lead to receptor downregulation, a common termination signal of canonical RTK activation [76]. As this effect is independent of the major *H. pylori* virulence factors, the disclosure of the causative mechanism involved in EPHA2 activation, whether a bacterial component mimicking the ligand, the induction of EPHA2 clustering as a result of bacterial binding by a physical mechanism, or others, could provide important insights into *H. pylori* virulence, eukaryotic signaling interactions, and the development of novel therapies.

Given the contrasting roles of EPHA2 in different tumor models and the pleiotropic roles in which EPHA2 is involved, we determined the functional consequences of *H. pylori* EPHA2-targeting in gastric cells using RNA interference. We showed that EPHA2 induces cell–cell adhesion, mediates cell–collagen type I interactions through α2β1 integrin, and suppresses cell invasion *in vitro*. Overall, these results point to EPHA2 acting in a fashion compatible with that of an adhesion-like molecule, stressing its importance for epithelial monolayer integrity through cell–cell and cell–ECM interactions. The crosstalk between the EPHA2 receptor and integrins may be important not only for the inhibition of cell spreading and invasion, but also in cell–cell communication with epithelial cells and other cellular components of the stroma [77,78,79,80,81]. Furthermore, as globally observed in the angiogenesis array, in endothelial tube formation, and in CAM assays, EPHA2 promotes angiogenesis in response to *H. pylori* infection of gastric cells through the secretion or induction of angiogenic factors and through tumor cell–endothelium interactions. These results are consistent with those of other studies implicating EPHA2 and other EPH receptors as regulators of angiogenesis in both tumor and endothelial cells [22,82,83,84,85].

Our results strengthen a model in which the modulation of cellular functions by *H. pylori* via EPHA2 in gastric cells contribute to disease pathogenesis. This may apply to gastric carcinogenesis, where the loss of cell–cell and cell–matrix adhesion and increased invasion and angiogenesis are pivotal, but may also be important for *H. pylori* colonization and persistence, where angiogenesis is essential for nutrient supply, mucosal damage repair, and immune regulation of the infection.

Given the ability of *H. pylori* to interfere with the activation of RTKs, thereby affecting their dynamics, steady-state levels, and cellular functions, further dissection of the underlying host signaling pathways and bacterial factors involved in *H. pylori*–EPHA2 interactions is needed. In the context of gastric cancers associated with *H. pylori*, it would be important to address the crosstalk of EPHA2 with other RTKs and with other signaling pathways that are well-known drivers of carcinogenesis and tumor aggressiveness, such as EGFR, MET, and the WNT/β-catenin pathway [86,87,88]. Finally, it would be relevant to investigate the impact of *H. pylori* on the expression of RTKs in gastric cancer patients undergoing therapies with small molecules targeting these receptors. This information may be valuable to evaluate potential interference with the efficacy of RTK therapies and, consequently, its use as a means of patient stratification.

## Figures and Tables

**Table 1:** Angiogenic factors induced by *H. pylori* with a fold-change of ≥1.5 (siNS_I/siNS_U).

Pro-Angiogenic Factors	Anti-Angiogenic Factors
ANGPT1 (Angiopoietin-1)	PF4 (platelet factor 4)
ANGPT2 (Angiopoietin-2) *	PLG (angiostatin/plasminogen)
ARTN (Artemin)	THBS2 (thrombospondin 2)
CCL2 (C-C motif chemokine ligand 2, MCP-1)	VASH (vasohibin)
CSF2 (granulocyte-macrophage colony stimulating factor)	
CXCL8 (C-X-C motif chemokine ligand 8, IL-8)	
CXCL16 (C-X-C motif chemokine ligand 16)	
EGF (epidermal growth factor)	
EDN1 (endothelin-1)	
FGF1 (acidic fibroblast growth factor 1)	
FGF2 (basic FGF fibroblast growth factor 2)	
FGF-4 (fibroblast growth factor 4)	
GDNF (glial cell-derived neurotrophic factor)	
HBEGF (heparin binding EGF-like growth factor)	
IGFBP3 (insulin-like growth factor binding protein 3)	
IL1B (Interleukin 1 beta)	
PDGF-AB/-BB (platelet-derived growth factor subunit AB/BB)	
PLAU (plasminogen activator urokinase, uPa)	
PSPN (Persephin)	
VEGFA (vascular endothelial growth factor A)	
VEGFC (vascular endothelial growth factor C)	

* Pro-angiogenic in the presence of VEGF [56].

**Table 2:** Angiogenic genes altered in the context of EPHA2-silencing comparing infected with uninfected siEPHA2_I/siEPHA2_U for fold-changes of ≤0.5 and ≥1.5.

FC ^1^ ≤ 0.5	FC ^1^ ≥ 1.5
**Pro-Angiogenic**	**Pro-Angiogenic**
EGF	CCL2/MCP-1
HGF	CXCL8/IL8
IGFBP1	FGF7/KGF
IGFBP2	HBEGF
IL1B/IL1F2	PDGF-AA
NRG1B1/HRG1B1	PLAU/uPA
PTX3/TSG14	PRL
TYMP/PD-ECGF	VEGFA
**Anti-angiogenic factors**	
ADAMTSL1	
TIMP1	
TIMP4	
SERPINF1	

^1^ FC—Fold-Change. Abbreviations: ADAMTSL1 (ADAM metallopeptidase with thrombospondin type 1 motif 1), FGF7/KGF (fibroblast growth factor 7), IL1B (interleukin 1 beta), NRG1 (neuregulin 1 beta1), PRL (prolactin), PTX3 (pentraxin 3), TIMP1 (tissue inhibitor of metalloproteinases 1), TIMP4 (tissue inhibitor of metalloproteinase 4), and TYMP/PD-ECGF (platelet-derived endothelial cell growth factor). See Table 1 for other abbreviation names.

## Supplementary Materials

The following are available online at https://www.mdpi.com/2073-4409/9/2/513/s1. Supplementary Figure S1 and Supplementary Table S1.
