# Supplementary material for: Helicobacter Pylori Targets the EPHA2 Receptor Tyrosine Kinase in Gastric Cells Modulating Key Cellular Functions
Source: Cells. 2020 Feb 24;9(2):513. doi: 10.3390/cells9020513 (PMC7072728; doi:10.3390/cells9020513)
Supplement: Supplementary file 1 [file cells-09-00513-s001.pdf]

Supplementary Materials

Leite *et al.* *H. pylori* targets the EPHA2 receptor tyrosine kinase in gastric cells modulating key cellular functions

Supplementary Figure S1.

(a)

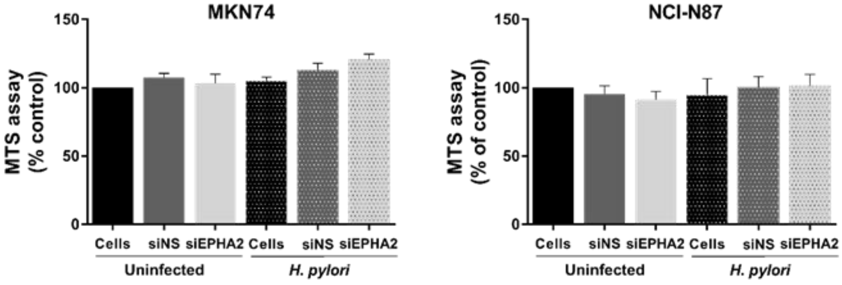

(b)

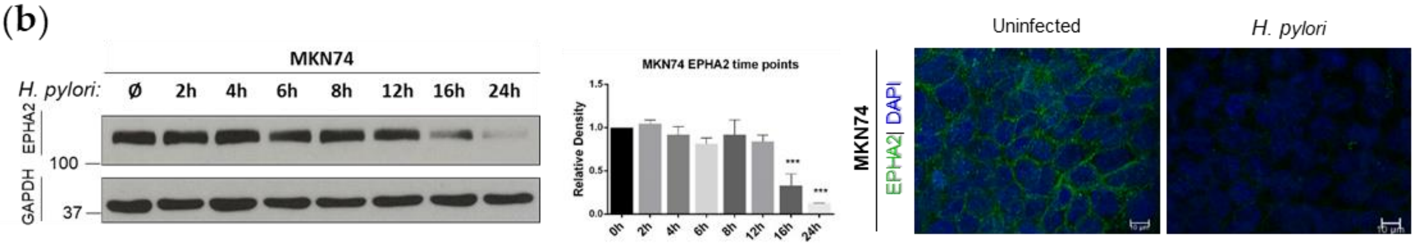

(c)

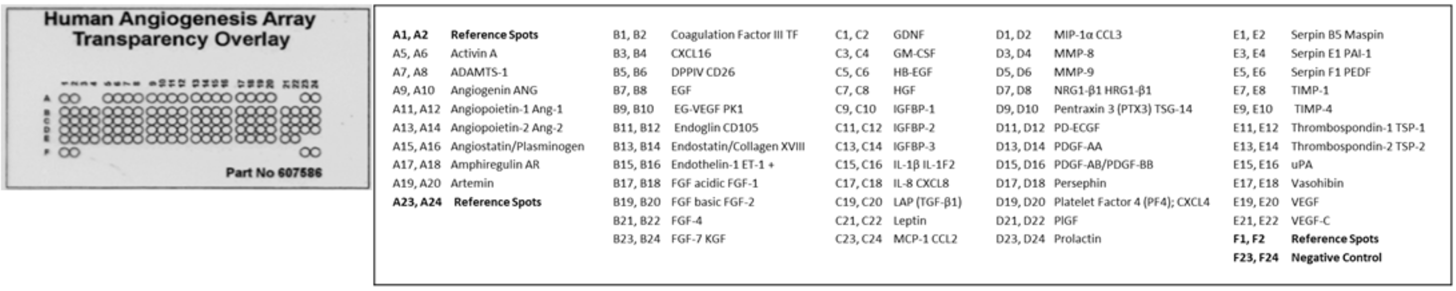

**A1, A2**	**Reference Spots**	B1, B2	Coagulation Factor III TF	C1, C2	GDNF	D1, D2	MIP-1α CCL3	E1, E2	Serpin B5 Maspin
A5, A6	Activin A	B3, B4	CXCL16	C3, C4	GM-CSF	D3, D4	MMP-8	E3, E4	Serpin E1 PAI-1
A7, A8	ADAMTS-1	B5, B6	DPPIV CD26	C5, C6	HB-EGF	D5, D6	MMP-9	E5, E6	Serpin F1 PEDF
A9, A10	Angiogenin ANG	B7, B8	EGF	C7, C8	HGF	D7, D8	NRG1-β1 HRG1-β1	E7, E8	TIMP-1
A11, A12	Angiopoietin-1 Ang-1	B9, B10	EG-VEGF PK1	C9, C10	IGFBP-1	D9, D10	Pentraxin 3 (PTX3) TSG-14	E9, E10	TIMP-4
A13, A14	Angiopoietin-2 Ang-2	B11, B12	Endoglin CD105	C11, C12	IGFBP-2	D11, D12	PD-ECGF	E11, E12	Thrombospondin-1 TSP-1
A15, A16	Angiostatin/Plasminogen	B13, B14	Endostatin/Collagen XVIII	C13, C14	IGFBP-3	D13, D14	PDGF-AA	E13, E14	Thrombospondin-2 TSP-2
A17, A18	Amphiregulin AR	B15, B16	Endothelin-1 ET-1 +	C15, C16	IL-1β IL-1F2	D15, D16	PDGF-AB/PDGF-BB	E15, E16	uPA
A19, A20	Artemin	B17, B18	FGF acidic FGF-1	C17, C18	IL-8 CXCL8	D17, D18	Persephin	E17, E18	Vasohibin
**A23, A24**	**Reference Spots**	B19, B20	FGF basic FGF-2	C19, C20	LAP (TGF-β1)	D19, D20	Platelet Factor 4 (PF4); CXCL4	E19, E20	VEGF
		B21, B22	FGF-4	C21, C22	Leptin	D21, D22	PlGF	E21, E22	VEGF-C
		B23, B24	FGF-7 KGF	C23, C24	MCP-1 CCL2	D23, D24	Prolactin	**F1, F2**	**Reference Spots**
								**F23, F24**	**Negative Control**

(d)

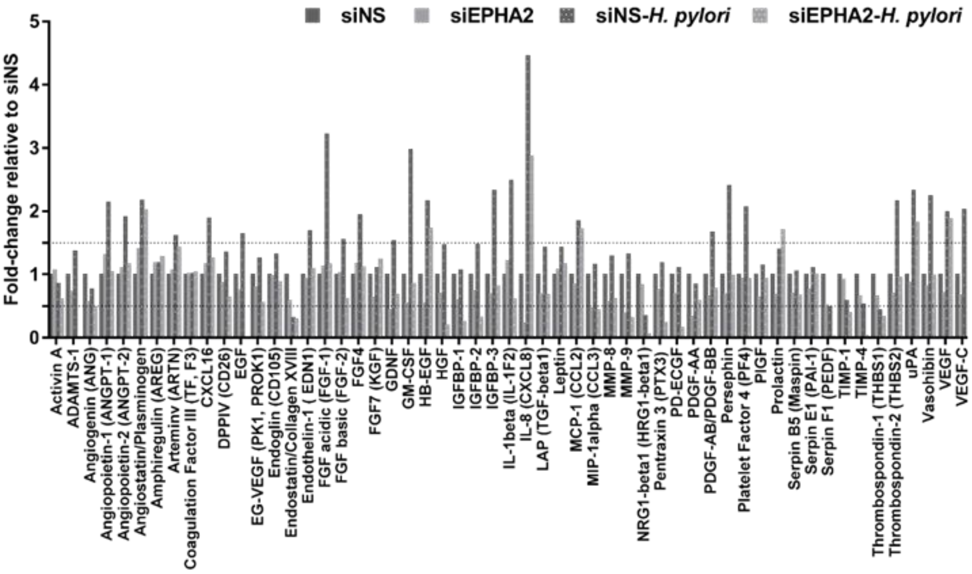

(e)

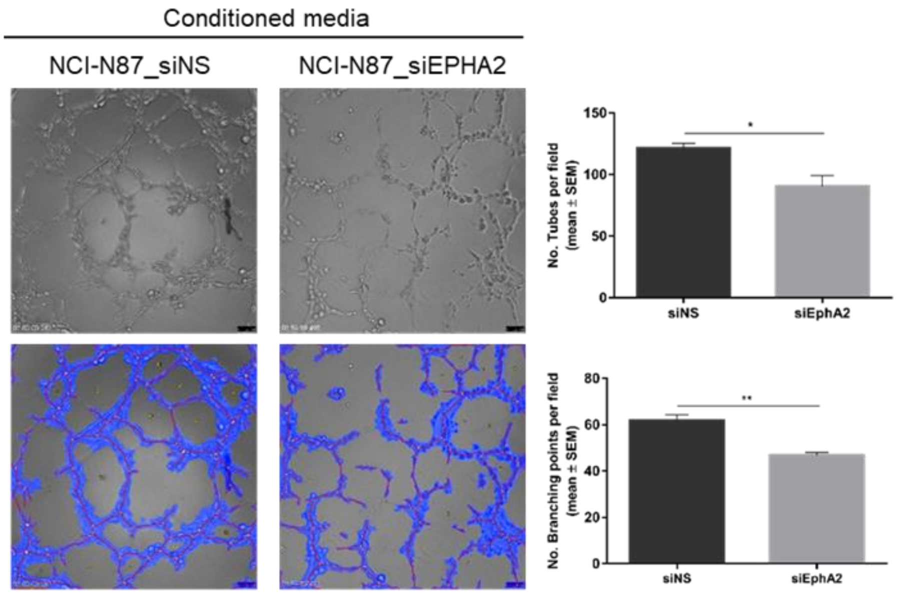

Supplementary Figure S1. (**a**) Cell viability of MKN74 and NCI-N87 cell lines evaluated by the CellTiter 96® AQueous One Solution Cell Proliferation Assay, in cells at confluence for 5 days in 96-well plates, uninfected or *H. pylori*-infected at MOI100 for 24 hrs (n=8). (**b**) Time-course experiment of *H. pylori* 26695 effect on EPHA2 protein expression in MKN74 cells by western blot and its densitometric quantification; immunofluorescence of EPHA2 (green) expression in MKN74 upon 16 h post-infection at MOI100 with *H. pylori* 26695 counterstained with DAPI for nuclei staining (blue) (scale bar: 10 µm; 63x original magnification). (**c**) Map of the Human Angiogenesis Array Proteome Profiler™ Array depicting the position of the duplicated spots for each of the fifty-five angiogenic-related factor used in this array and the corresponding name list (R&D Systems). (**d**) Relative expression in fold-change calculated from the siNS-normalized average pixel density of the duplicated spots for each angiogenic-related protein in the array. (**e**) Representative micrographs of the *in vitro* capillary-like structures formed by Human Umbilical Vein Endothelial Cells (HUVECs) upon treatment with conditioned medium from NCI-N87 cells transfected with a non-silencing siRNA (siNS) or with a siRNA for EPHA2 (siEPHA2), 5 h post-seeding in Matrigel coated wells, and corresponding automatically analysis using the WimTube software (scale bar: 100 µm; original magnification, ×100). Quantification of the number of tubes and branching points per microscopic field from three independent experiments, using the WimTube software. Data is presented as mean ± SEM (n=3). Unpaired Student's t-test; * p< 0.01; **p< 0.001.

Supplementary Table 1

	NS_U	siEphA2_U	NS_I	siEphA2_I
Activin A (A5, A6)	8.187.095,90	8.737.071,04	7.017.784,62	4.974.678,98
ADAMTS-1 (A7, A8)	437.201,63	321.170,49	597.530,86	-113.026,52
Angiogenin ANG (A9, A10)	2.113.315,07	1.202.032,99	1.634.430,70	1.031.402,25
Angiopoietin-1 Ang-1 (A11, A12)	654.602,40	856.003,35	1.401.673,61	680.255,12
Angiopoietin-2 Ang-2 (A13, A14)	942.470,01	1.046.800,32	1.799.658,10	1.099.420,38
Angiostatin/Plasminogen (A15, A16)	458.126,67	645.205,39	993.879,27	927.190,24
Amphiregulin AR (A17, A18)	20.880.917,09	24.857.954,52	24.784.044,66	26.652.958,49
Artemin (A19, A20)	1.000.051,07	1.065.667,28	1.614.306,31	1.429.644,96
Coagulation Factor III TF (B1, B2)	29.381.783,48	29.795.737,35	29.779.342,92	30.565.884,87
CXCL16 (B3, B4)	3.087.081,59	3.617.902,14	5.840.676,00	3.875.783,16
DPPIV CD26 (B5, B6)	6.248.764,44	5.419.084,42	8.452.216,42	3.993.031,10
EGF (B7, B8)	696.217,48	524.995,92	1.142.964,20	-1.135,95
EG-VEGF PK1 (B9, B10)	3.380.264,38	2.697.811,67	4.263.416,61	1.886.494,94
Endoglin CD105 (B11, B12)	15.492.317,65	15.124.075,76	20.475.162,18	13.736.796,09
Endostatin/Collagen XVIII (B13, B14)	15.945.835,13	9.368.321,59	5.084.219,80	4.771.137,23
Endothelin-1 ET-1 (B15, B16)	1.390.695,04	1.312.234,40	2.356.349,52	1.512.148,00
FGF acidic FGF-1 (B17, B18)	891.442,24	1.003.474,17	2.867.554,53	1.043.172,51
FGF basic FGF-2 (B19, B20)	699.520,99	720.444,23	1.082.565,82	431.400,41
FGF-4 (B21, B22)	687.514,41	810.406,53	1.332.842,46	772.308,59
FGF-7 KGF (B23, B24)	1.017.852,56	653.467,60	1.134.399,52	1.260.133,63
GDNF (C1, C2)	4.013.087,43	1.783.469,41	6.154.817,23	2.734.080,98
GM-CSF (C3, C4)	1.215.456,32	667.379,58	3.625.684,79	1.044.174,94
HB-EGF (C5, C6)	11.850.745,10	6.551.723,77	25.619.231,49	20.462.726,56
HGF (C7, C8)	897.753,94	631.435,05	1.319.480,93	182.140,99
IGFBP-1 (C9, C10)	2.113.314,25	1.277.835,91	2.256.326,33	557.735,09
IGFBP-2 (C11, C12)	1.158.010,05	860.743,03	1.711.857,44	382.182,88
IGFBP-3 (C13, C14)	1.356.566,83	942.466,27	3.158.924,58	1.118.062,55
IL-1β IL-1F2 (C15, C16)	478.491,08	584.584,25	1.190.383,31	290.768,12
IL-8 CXCL8 (c17, C18)	6.797.899,02	1.557.136,17	30.332.921,74	19.488.293,91
LAP (TGF-β1) (C19, C20)	2.561.975,17	1.758.885,69	3.651.708,26	1.743.194,87
Leptin (C21, C22)	478.594,24	517.694,81	682.117,04	561.170,78
MCP-1 CCL2 (C23, C24)	638.179,60	544.673,38	1.180.010,42	1.095.265,37
MIP-1α CCL3 (D1, D2)	1.396.011,87	661.535,05	1.624.155,02	607.720,18
MMP-8 (D3, D4)	1.065.482,06	606.150,02	1.376.621,67	657.249,02
MMP-9 (D5, D6)	2.295.606,26	894.471,66	3.031.112,19	744.640,39
NRG1-β1 HRG1-β1 (D7, D8)	3.916.623,61	3.303.729,11	1.380.935,09	238.515,63
Pentraxin 3 (PTX3) TSG-14 (D9, D10)	1.430.358,44	1.088.567,87	1.703.231,29	347.911,71
PD-ECGF (D11, D12)	1.779.054,10	1.223.574,97	1.968.520,73	286.900,91
PDGF-AA (D13, D14)	5.256.720,34	1.781.223,19	4.476.620,96	3.096.039,70
PDGF-AB/PDGF-BB (D15, D16)	960.909,09	634.648,89	1.607.186,05	752.587,89
Persephin (D17, D18)	1.583.759,81	1.092.199,28	3.813.362,41	1.570.338,81
Platelet Factor 4 (PF4) CXCL4 (D19, D20)	817.015,17	764.431,11	1.687.707,27	771.765,35

PlGF (D21, D22)	1.163.110,34	746.933,20	1.334.423,97	1.092.976,35
Prolactin (D23, D24)	427.760,60	291.471,10	597.222,50	730.302,31
Serpin B5 Maspin (E1, E2)	7.851.022,86	5.529.969,14	8.243.269,11	5.262.309,71
Serpin E1 PAI-1 (E3, E4)	15.686.962,65	12.082.501,21	17.427.182,02	15.737.388,30
Serpin F1 PEDF (E5, E6)	1.734.182,81	898.055,31	853.768,38	40.822,08
TIMP-1 (E7, E8)	29.940.067,90	27.744.039,70	17.522.035,78	11.851.236,65
TIMP-4 (E9, E10)	2.860.408,71	1.891.964,55	1.521.908,04	78.152,44
Thrombospondin-1 TSP-1 (E11, E12)	23.454.225,98	15.462.898,67	10.431.803,76	8.063.253,37
Thrombospondin-2 TSP-2 (E13, E14)	1.026.021,53	719.349,32	2.212.276,74	981.185,23
Upa (E15, E16)	636.404,74	557.835,48	1.481.878,18	1.161.956,96
Vasohibin (E17, E18)	719.005,38	591.530,70	1.611.468,05	709.979,56
VEGF (E19, E20)	10.985.846,97	7.771.303,40	21.879.697,36	20.620.272,89
VEGF-C (E21, E22)	465.069,39	312.957,18	945.938,40	411.634,91
